# An integrated multi-omics study of key mediators and therapeutic targets for doxorubicin-induced atrial fibrillation

**DOI:** 10.1371/journal.pone.0353143

**Published:** 2026-07-09

**Authors:** Zhenli Li, Sihan Liu, Jing He, Tenghui Wang, Jingtao Ma

**Affiliations:** 1 Department of Cardiology, The Fourth Hospital of Hebei Medical University (the Tumor Hospital of Hebei Province), Shijiazhuang, Hebei, People’s Republic of China; 2 The Fourth Hospital of Hebei Medical University (the Tumor Hospital of Hebei Province), Shijiazhuang, Hebei, People’s Republic of China; 3 Department of Cardiology, Peking University International Hospital, No.1 Life Park Road, Zhongguancun Life Science Park, Changping District, Beijing, People’s Republic of China; 4 School of Basic Medicine, Hebei Medical University, Shijiazhuang, Hebei, People’s Republic of China; Sichuan University, CHINA

## Abstract

**Background:**

Doxorubicin (DOX), a widely used chemotherapeutic agent for cancer patients, is associated with a significant risk of inducing atrial fibrillation (AF), a serious cardiac complication that impairs patient prognosis. However, the specific molecular and cellular mechanisms linking DOX cardiotoxicity to AF pathogenesis remain poorly understood.

**Methods:**

Following processing pharmacovigilance analysis of DOX-related AF events, we employed an integrative multi-omics strategy. Differentially expressed genes (DEGs) were first identified from the atrial transcriptomic dataset. Network toxicology was used to predict DOX targets, which were intersected with AF-related genes and DEGs to identify candidate targets. Functional analyses and protein-protein interaction network analysis was applied to pinpoint hub genes. Their predictive performance was validated in independent datasets. Gene set enrichment analysis (GSEA) and immune infiltration profiling (CIBERSORT) were conducted to elucidate biological functions and immune context. Molecular docking simulations validated direct interactions between DOX and selected proteins. Finally, single-cell RNA sequencing (scRNA-seq) analysis resolved the cell-type-specific expression patterns of key targets.

**Results:**

Functional analyses implicated the candidate genes in critical pathways. 5 hub genes were further selected from candidate genes using the MCC algorithm. Among 5 hub genes, we identified and validated the combination of *CCR2*, *PDE5A*, and *CXCR2* showed high predictive accuracy for AF (mean AUC = 0.87), with identifying and validating *CCR2* and *PDE5A* significantly and differentially expressed. GSEA linked *CCR2* and *PDE5A* showed different pathways. Immune infiltration analysis revealed significant alterations in macrophages, monocytes, and T cell subsets in AF tissues. Molecular docking confirmed stable, high-affinity binding between DOX and both *CCR2* and *PDE5A* (binding energy < −7 kcal/mol). Crucially, scRNA-seq analysis demonstrated that *CCR2* and *PDE5A* were differentially expressed in atrial macrophages and fibroblasts respectively.

**Conclusion:**

This study suggests that *CCR2* and *PDE5A* may serve as central mediators and potential therapeutic targets for DOX-induced AF, though these findings require experimental validation.

## 1. Introduction

Atrial fibrillation (AF), known as a common type of cardiac arrhythmia, is characterize by chaotic electrical activity within the atria, with a global age-related prevalence of 0.62% and an incidence ranging from 2% to 16% during cancer treatment [1,2]. AF impairs atrial contraction and diminishes quality of life, thereby increasing the risk of cardiovascular disorders like stroke and heart failure, which ultimately lead to an elevated mortality [3]. Anthracyclines are widely used chemotherapeutic agents for cancer patients, which are related to cancer therapeutics-related cardiac dysfunction [4]. Amioka et al. observed 6% newly developed AF among 249 lymphoma patients after anthracycline chemotherapy [5]. As a common chemotherapeutic agent and a type of Anthracyclines, doxorubicin (DOX) is effective against a broad spectrum of cancers, including breast cancer and lymphoma [6]. Nevertheless, its therapeutic application is significantly restricted by its dose-dependent cardiotoxic effects. Notably, a rate of 10.3% of patients who received DOX therapy developed AF was observed in recent studies, which is a DOX-induced acute cardiotoxicity [3,7]. Moreover, a study revealed AF comprised 30.4% of total DOX-induced cardiotoxicity among patients treated for non-Hodgkin lymphoma with first-line DOX-based chemotherapy [8]. Therefore, explore the potential gene targets for DOX-induced AF holds great clinical significance for cancer patients receiving the DOX therapy.

There are several studies, which have explored the mechanism of DOX-induced cardiotoxicity [9]. Multi-cells, including cardiomyocytes, endothelial cells, smooth muscle cells, and immune cells has been demonstrated being involved in the cardiotoxicity, with underlying mechanisms including oxidative stress, impaired mitochondrial function, endothelial injury, apoptosis, autophagy, pyroptosis, and ferroptosis [10,11]. However, the study exploring the mechanism of DOX-induced AF is still rare for the complexity of DOX-induced cardiotoxicity. Regarding the development of AF, electrical and calcium remodelling in atrial cardiomyocytes, stress signalling pathways such as JUN N-terminal kinases (JNKs), NLRP3 inflammasomes, JNK2, and mitochondrial dysfunction are the mechanisms/pathophysiology [12]. Moreover, Zhang et al. elucidated oxidative stress and pyroptosis in DOX-induced heart failure and AF [13].

Though there exists a clinical significance of DOX-induced AF, direct studies establishing the mechanistic basis of the DOX-induced AF are still limited. For example, how the key cells within atria interact with AF progression. In this work, transcriptomics and network toxicology were utilized to identify biomarkers associated with DOX-induced AF. Subsequently, we applied a battery of bioinformatics tools to delineate the specific mechanisms by which these biomarkers promote AF initiation. Furthermore, using single-cell data, we validated their expression patterns in relevant cell populations. The results offered fresh theoretical grounding and perspectives for understanding AF etiology and informing personalized therapeutic strategies, thereby bridging a critical gap in current AF biomarker research, which has overlooked molecular targets connecting DOX cardiotoxicity to specific cell activity.

## 2. Methods

### 2.1. Overall study design and data sources

**Fig 1:**
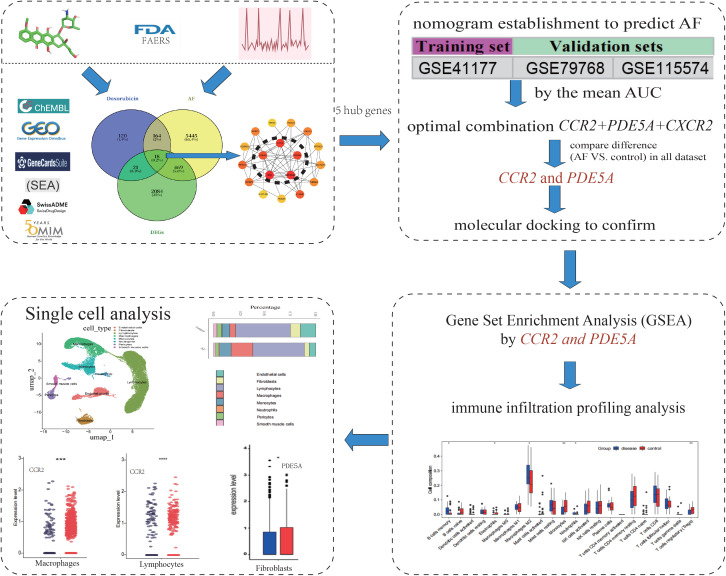
The overall design of the present study.

As shown in Fig 1, the overall study design is presented as a flow chart. The FDA Adverse Event Reporting System (FAERS) database, utilized in the present study and available at https://www.fda.gov/drugs/surveillance/fdas-adverse-event-reporting-system-faers, comprises adverse event reports, medication error reports, and product quality complaints submitted to the FDA. Adverse events and medication errors within this system are coded using terminology from the Medical Dictionary for Regulatory Activities (MedDRA). The GSE41177 dataset from GEO database, including 32 AF and 6 sinus rhythm (SR) atrial appendage samples, was served as a training group to identify hub genes when combining the result of the network toxicology analysis. The following two atrial tissue transcriptomic datasets functioned as validation sets in our study: (1) GSE115574 dataset, including 28 AF and 31 SR atrial samples from 15AF patients and 15 SR patients; (2) GSE79768 dataset, comprising 14 AF and 12 atrial specimens. The GSE197518 dataset from the GEO database was retrieved to work as the Single-cell dataset, consisting of 7 AF samples from left atrial tissues collected from 7 patients with persistent AF undergoing surgical mitral valve repair and 5 SR samples from 5 control subjects. The databases for network toxicology analysis were detailed at the section 2.4. This study did not require ethical approval or informed consent because it involved only the analysis of publicly available, de-identified data from three sources: FAERS, GEO, and publicly available databases for network pharmacology. No human subjects were directly enrolled, no animal experiments were performed, and no privacy-sensitive information was accessed.

### 2.2. Data processing and signal mining of FAERS database

We performed a pharmacovigilance analysis of adverse events associated with DOX using data from the FAERS. Reports involving DOX were extracted from the FAERS public dashboard for the period spanning Q1 2004 to Q3 2025. Duplicate entries were removed to enhance data reliability according to FDA-recommended data cleaning steps. Adverse event cases related to the DOX were identified using the Medical Dictionary for Regulatory Activities (MedDRA version 26.0) and were defined based on the preferred term named “ATRIAL FIBRILLATION”. To evaluate the strength of potential safety signals, we computed commonly used disproportionality measures, including the reporting odds ratio (ROR) and information component (IC). A potential safety signal was defined when the lower limit of the 95% confidence interval (CI) of the ROR exceeded 1 (ROR025 > 1) and the number of individual case safety reports (n) was ≥ 3. For the Information Component (IC), a positive signal was considered when the lower bound of the 95% credible interval of the IC exceeded 0 (IC025 > 0), which represents a statistically significant increase in reporting probability based on the Bayesian confidence propagation neural network (BCPNN) approach. Formulas and interpretations for these metrics in the previous study [14]. Statistically significant signals were further examined with chi-square tests. The entire data extraction and analytical workflow adhered to established pharmacovigilance methodology and standard operating procedures, as outlined in S1 Fig. Moreover, The FAERS database provides the Indication Preferred Term (INDI_PT) field, which records the primary indication for the drug reported in each adverse event case, coded using the MedDRA dictionary. For this study, we systematically classified and aggregated these INDI_PT terms to determine the distribution of primary tumor types among the included reports.

### 2.3. Differentially expressed gene analysis between AF and SR patients

In the present study, GSE41177 dataset from GEO database was utilized to obtain the differentially expressed genes (DEGs). The screening parameter for identification of DEGs was as follows: (1) adjusted P value<0.05; (2) |log_2_ fold change (log_2_ FC)| > 0.5. A volcano plot was utilized to visualize the DEGs. According to the order of their log_2_ FC, the top 10 significant genes were also annotated with a heatmap displaying the expression patterns of the top 15 most upregulated and downregulated genes.

### 2.4. Identification of molecular targets in DOX-induced AF

The molecular structure and simplified molecular input line entry system (SMILES) notation of DOX were obtained from ChemSpider (https://www.chemspider.com/) [15]. To identify potential molecular targets of DOX, we conducted comprehensive target prediction analyses using multiple databases. Firstly, putative DOX-related targets were predicted using the ChEMBL (https://www.ebi.ac.uk/chembl/) [16], SwissTargetPrediction (http://www.swisstargetprediction.ch/) [17], and Similarity Ensemble Approach (SEA, https://sea.bkslab.org/) [18] databases using SMILES or “doxorubicin” as the key word. To identify therapeutic molecular targets of AF, we consulted GeneCards (https://www.genecards.org) [19] and Online Mendelian Inheritance in Man (OMIM, http://www.omim.org/) [20] databases to extract them with the keyword “atrial fibrilation”. Accordingly, target protein names were standardized according to UniProt nomenclature (https://www.uniprot.org/) [21]. Through intersection analysis of DOX-related targets, AF-associated genes, and DEGs of AF in the section 2.2, we identified candidate mechanistic targets involved in DOX-induced AF after removing redundant entries of target genes.

### 2.5. Functional annotation and pathway enrichment analysis and hub genes identification

To explore the biological functions and signaling pathways of the candidate genes selected in Section 2.3, we performed Gene Ontology (GO) [22] and KEGG [23] pathway enrichment analyses with a significance threshold of both p-value and adjusted p-value < 0.05. To delineate the molecular interaction landscape, we constructed a protein-protein interaction (PPI) network using the STRING database (https://cn.string-db.org/) [24] with the identified candidate targets. A minimum interaction confidence score threshold of 0.15 was selected to capture enough interactions while maintaining network comprehensiveness. Cytoscape (version 3.7.1) was then utilized for visualizing the analysis result of the DOX-induced AF network. Assisting by CytoHubba plugin [25], Hub genes were identified with the Maximal Clique Centrality (MCC) algorithm.

### 2.6. Prediction performance of the hub genes to predict AF

Hub genes, including *CCR2*, *PDE5A*, *CXCR2*, *HSP90AB1*, and *KDR*, were utilized to construct 31 predictive combinations from single-gene to penta-gene forms. The GSE41177 dataset served as the training set, with GSE79768 and GSE115574 as external validation sets to evaluate prediction performance. AUC (Area Under the ROC Curve) was calculated for each combination across all datasets, and the mean of AUCs was used to assess overall prediction accuracy. Nomograms were built to visualize the correlation between gene expression-derived points and AF mortality risk, while Decision Curve Analysis (DCA) was performed to determine clinical net benefits at different threshold probabilities. A calibration plot was generated to compare the observed AF risk with the predicted probability, verifying the consistency of the prediction model. Additionally, gene expression levels were compared between control and disease groups in the three datasets to validate differential expression of the genes in the selected model.

### 2.7. Gene set enrichment analysis (GSEA) and Immunoinfiltration analysis

To elucidate the underlying biological functions and pathways, we performed Spearman correlation analysis between *CCR2*/*PDE5A* and all other genes in the GSE41177 dataset. The resulting correlation coefficients were ranked before being used for GSEA with the following significance thresholds: P < 0.05, ∣NES∣ > 1, and FDR < 0.25. To assess immune cell infiltration, we estimated the relative abundances of multiple immune cell types using the CIBERSORT R package and compared their profiles between AF and SR samples (GSE115574). Immune cell subsets showing statistically significant differences between the AF and SR groups (p < 0.05) were also identified. Finally, Pearson correlation analysis was applied to evaluate associations between these differentially infiltrated immune cell subsets respectively.

### 2.8. Molecular docking analysis

To validate whether *CCR2* and *PDE5A* could serve as potential targets of DOX to induce AF, molecular docking analyses were performed. Protein structures corresponding to genes were collected from the RCSB Protein Data Bank (PDB, https://www.rcsb.org/) [26]. The 3D structure of DOX was downloaded from PubChem (https://pubchem.ncbi.nlm.nih.gov) [27]. Following format conversion to PDB, molecular docking was carried out using AutoDock Vina to screen binding poses and evaluate affinity. The most stable conformation, determined by minimal binding energy, was further examined in PyMOL (version 3.0.3) and LigPlus (version 2.2.4) to characterize interaction patterns. Specifically, binding affinities were classified as weak (−4.25 to −5.00 kcal/mol), moderate (−5.00 to −7.00 kcal/mol), or strong (below −7.00 kcal/mol) [28].

### 2.9. Single-cell data analysis

scRNA-seq data (GSE197518) from the GEO database were pre-processed using the Seurat v5 R package. The steps are as follows: (1) Quality control: Gene count between 200 and 5000 per cell, and mitochondrial ratio under 20%. (2) Dimensionality reduction: Clustering was performed with the “FindClusters” function at 10 PCs and a resolution of 0.4. UMAP algorithm was applied to simplify the data structure and reveal the underlying cell population characteristics. (3) Cell annotation: Cell types were identified and annotated based on marker gene information from relevant literatures [29,30]. (4) Differences illustration of proportions of cell sub-types between the AF and SR groups. (5) Identification of key cell types: The differences of expression level of CCR2 and PDE5A in cell sub-types and key cell types were identified between the AF and SR groups by performing the Wilcoxon test.

## 3. Result

### 3.1. FAERS database analysis and Identification of DEGs and potential target genes for DOX-induced AF

**Fig 2:**
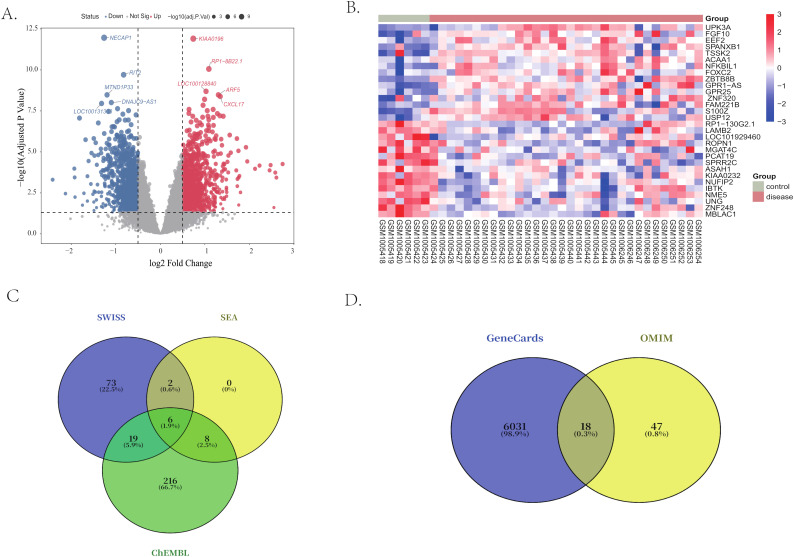
Identification of differentially expressed genes (DEGs) and potential targets for doxorubicin (DOX) and atrial fibrillation (AF). (A) Volcano plot visualizing DEGs between AF and sinus rhythm (SR) atrial tissues in the GSE41177 dataset. (B) Heatmap showing the top 15 most upregulated and downregulated DEGs. (C) Venn diagram illustrating the overlap of DOX-related targets predicted by three databases: ChEMBL, SwissTargetPrediction, and SEA. (D) Venn diagram illustrating the overlap of AF-related genes retrieved from GeneCards and OMIM databases.

**Fig 3:**
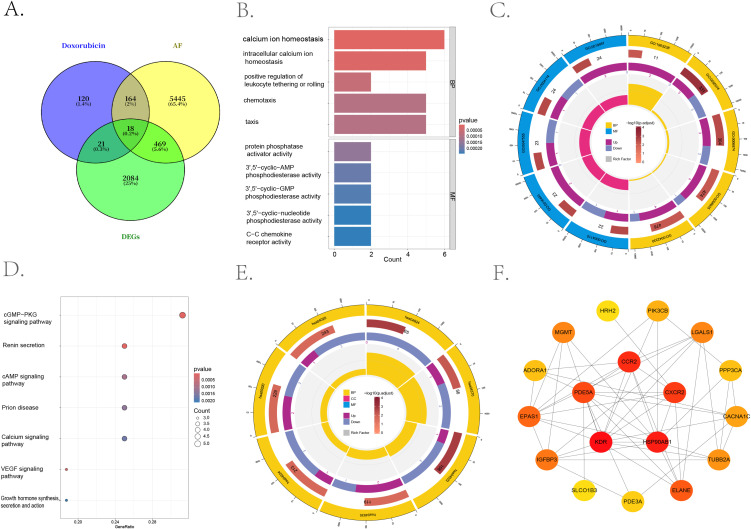
Functional annotation, pathway enrichment, and hub gene identification of candidate targets. (A) Venn diagram showing the intersection of DOX-related targets, AF-related genes, and DEGs. (B–C) Gene Ontology (GO) enrichment analysis of candidate targets. (D–E) Kyoto Encyclopedia of Genes and Genomes (KEGG) pathway enrichment analysis of candidate targets. (F) Protein-protein interaction (PPI) network of candidate targets visualized by Cytoscape. Hub genes (*CCR2*, *PDE5A*, *CXCR2*, *HSP90AB1*, *KDR*) identified by the Maximal Clique Centrality (MCC) algorithm are in the center.

Regarding the clinical relevance of DOX in AF, it showed a statistically significant signal for AF-AEs (ROR = 2.04, 95% CI: 1.50–2.77), supported by other disproportionality metrics (IC025 = 0.54) (S1 Fig). Moreover, the distribution of primary tumor types, classified by INDI_PT in the FAERS database, was dominated by lymphoma (34%), followed by missing/unknown indications (17%), breast cancer (12%), sarcoma (11%), ovarian cancer (7%), other rare tumor types (7%), myeloma (5%), leukemia (5%), and liver cancer (2%). A total of 4226 DEGs were identified through threshold-based screening, including 2178 up-regulated genes and 2048 down-regulated genes in the disease group (Fig 2A and Fig 2B). The SMILES notation for DOX is “COc1cccc2c1C(=O)c1c(O)c3c(c(O)c1C2=O)C[C@@](O)(C(=O)CO)C[C@@H]3O[C@H]1C[C@H](N)[C@H](O)[C@H](C)O1”. The ChEMBL, SwissTargetPrediction, and SEA databases identified 249, 80, and 16 potential targets, respectively. After removing duplicated targets, 324 toxicity targets were identified (Fig 2C and S1 Table). The Genecards and OMIM databases contained 6049 and 65 AF-related target genes (Fig 2D), respectively. Subsequently, 6096 AF-related target genes were obtained by removing duplicated genes (S1 Table). Through intersection analysis of DOX targets, AF-associated genes, and DEGs, after redundant entries were removed, candidate mechanistic targets involved in DOX-induced AF were successfully obtained (Fig 3A).

### 3.2. Functional annotation, pathway enrichment analysis, and hub genes identification

GO and KEGG pathway enrichment analyses were performed for the candidate genes, with a significance threshold of P value and adjusted P value < 0.05. The results showed that these candidate genes were significantly enriched in biological processes such as calcium ion homeostasis, intracellular calcium ion homeostasis, positive regulation of leukocyte tethering or rolling, and chemotaxis. For molecular functions, enrichment was observed in protein phosphatase activator activity, 3’,5’-cyclic-AMP phosphodiesterase activity, 3’,5’-cyclic-GMP phosphodiesterase activity, 3’,5’-cyclic-nucleotide phosphodiesterase activity, and C-C chemokine receptor activity (Fig 3B, Fig 3C, and S2 Table). KEGG pathway enrichment indicated involvement in the cGMP-PKG signaling pathway, Renin secretion, cAMP signaling pathway, Calcium signaling pathway, VEGF signaling pathway, and Growth hormone synthesis, secretion and action, among others (Fig 3D and Fig 3E, S2 Table). The PPI network of candidate targets was constructed using the STRING database and visualized with Cytoscape 3.7.1. Using the CytoHubba plugin and MCC algorithm, hub genes, including *CCR2*, *PDE5A*, *CXCR2*, *HSP90AB1*, and *KDR*, were identified (Fig 3F, S3 Table).

### 3.3. Prediction performance of hub genes for AF

**Fig 4:**
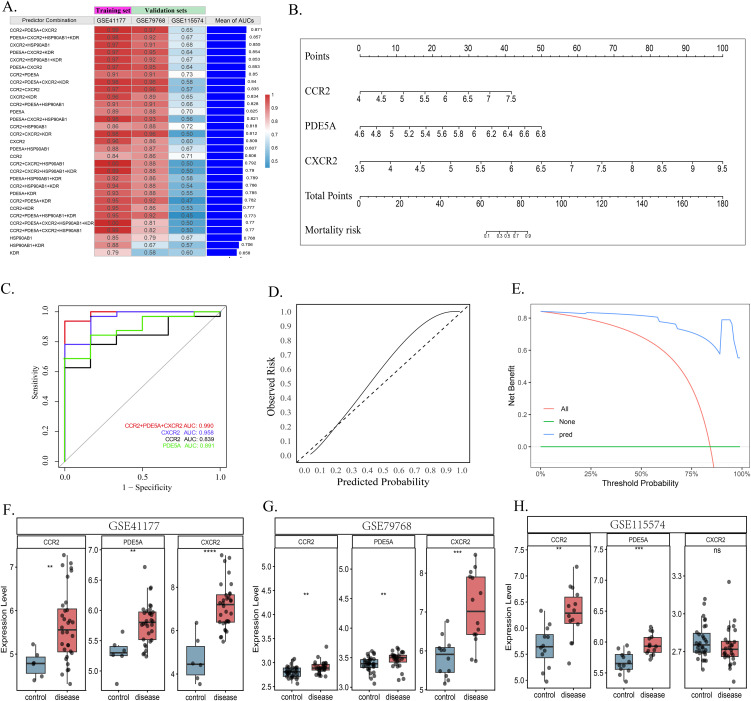
Predictive performance of hub gene combinations for Atrial Fibrillation (AF). (A) Bar plot showing the mean Area Under the Curve (AUC) of 5 hub gene combinations (single-gene to penta-gene) across training (GSE41177) and validation (GSE79768, GSE115574) datasets. The *CCR2* + *PDE5A*+*CXCR2* combination achieved the highest mean AUC (0.87). (B) Nomogram for predicting AF risk based on the *CCR2* + *PDE5A*+*CXCR2* combination. (C-E) ROC curves, calibration plot and decision curve analysis (DCA) of the *CCR2* + *PDE5A*+*CXCR2* model. (F–G) Box plots comparing *CCR2* (F), *PDE5A* (G) and *CXCR2* (H) expression levels between AF and Sinus Rhythm (SR) groups in training and validation datasets. ns = non-significant, **P < 0.01, ***P < 0.001.

A total of 31 predictive combinations (single-gene to penta-gene) were constructed using the identified hub genes. The *CCR2* + *PDE5A*+*CXCR2* combination achieved the highest mean AUC value of 0.87 by considering the comprehensive performance in both training and validation sets (Fig 4A and Fig 4C). A nomogram was constructed to visualize the AF risk model and a calibration plot showed good consistency between the observed AF risk and the predicted probability, validating the reliability of the prediction model (Fig 4B and Fig 4D). DCA demonstrated that the predictive models offered significant clinical net benefits across different threshold probabilities (Fig 4E). Furthermore, comparison of gene expression levels between control and AF groups in both training and validation sets further confirmed the significance of genes in the best-performed model, supporting the potential of *CCR2* and *PDE5A* as predictive biomarkers (Fig 4F and Fig 4G). As for *CXCR2*, not all gene expression levels between control and AF groups in both training and validation sets were observed significantly different (Fig 4H). Though not being selected into the best-performed model, *HSP90AB1* also owned a significantly high expression level in both 3 datasets (S2 Fig).

### 3.4. Gene set enrichment analysis (GSEA) and Immunoinfiltration analysis

**Fig 5:**
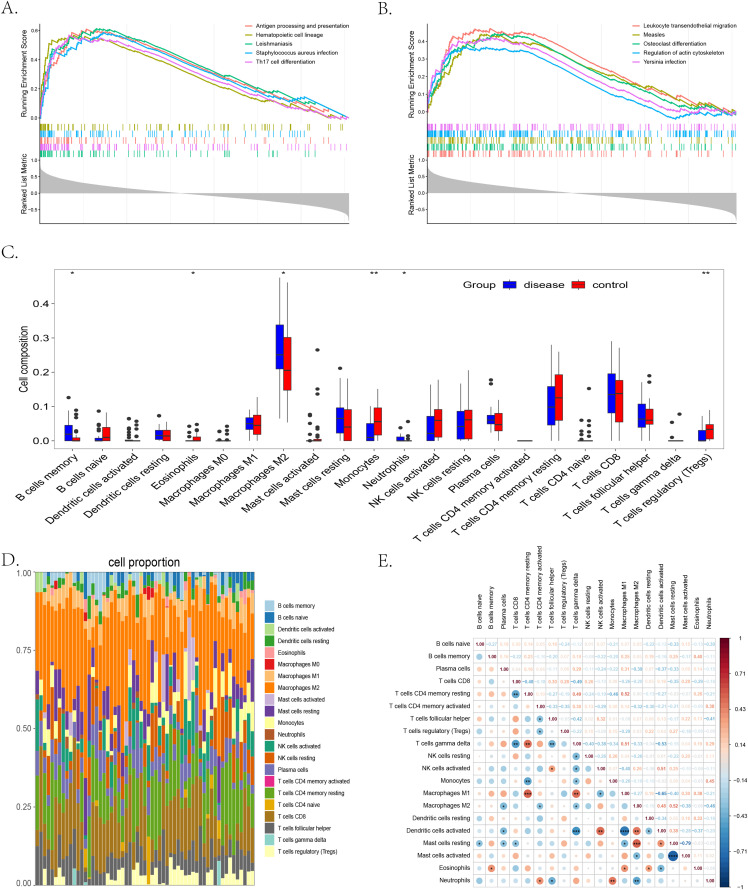
Gene Set Enrichment Analysis (GSEA) and immune infiltration profiling. (A–B) GSEA plots showing pathways enriched in genes positively correlated with *CCR2* (A) and *PDE5A* (B) in the GSE41177 dataset. (C) Box plots comparing the relative abundance of infiltrated immune cell subsets between atrial fibrillation (AF) (disease) and sinus rhythm (SR) (control) groups. (D) Bar plot showing the relative proportion of all immune cell types in AF and SR samples. (E) Correlation matrix of differentially infiltrated immune cell subsets. ns = non-significant, **P < 0.01, ***P < 0.001.

*CCR2* is primarily involved in notable pathways, which include antigen processing and presentation, hematopoietic cell lineage, leishmaniasis, staphylococcus aureus infection, and Th17 cell differentiation pathways (Fig 5A). Moreover, *PDE5A* is primarily involved in notable pathways, which include leukocyte transendothelial migration, measles, osteoclast differentiation, regulation of actin cytoskeleton, Yersinia infection pathways (Fig 5B). Following the algorithm’s confidence evaluation criteria, valid immune cell content data were retained from all samples (Fig 5D). After rigorous statistical testing, 6 immune cell types with significant differences were identified: B cells memory, eosinophils, M2 macrophages, monocytes, neutrophils, and T cells regulatory (Tregs) cells (Fig 5C). The correlation matrix shows correlation coefficients between different immune cell types (Fig 5E).

### 3.5. Results of Molecular docking validation between DOX and core targets

**Fig 6:**
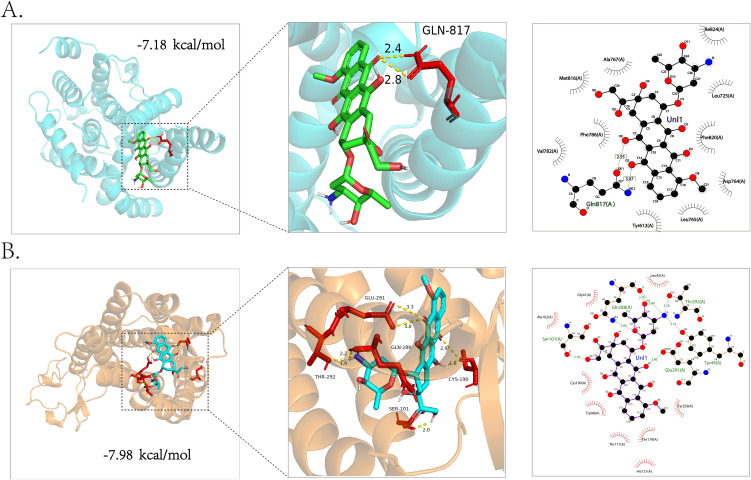
Molecular docking validation of DOX binding to *CCR2* and *PDE5A.* (A) Three-dimensional (3D) structure of the DOX-*CCR2* complex. (B) 3D structure of the DOX-*PDE5A* complex.

To verify the potential binding interactions between DOX and the identified core genes (*CCR2* and *PDE5A*), molecular docking assays were conducted. The outcomes revealed robust binding affinities between DOX and these two target proteins, with the minimum binding energies persistently falling below −7 kcal/mol. This finding suggests that the molecular interactions between DOX and the target proteins are stable and occur spontaneously (Fig 6).

### 3.6. The findings of single-cell analysis

**Fig 7:**
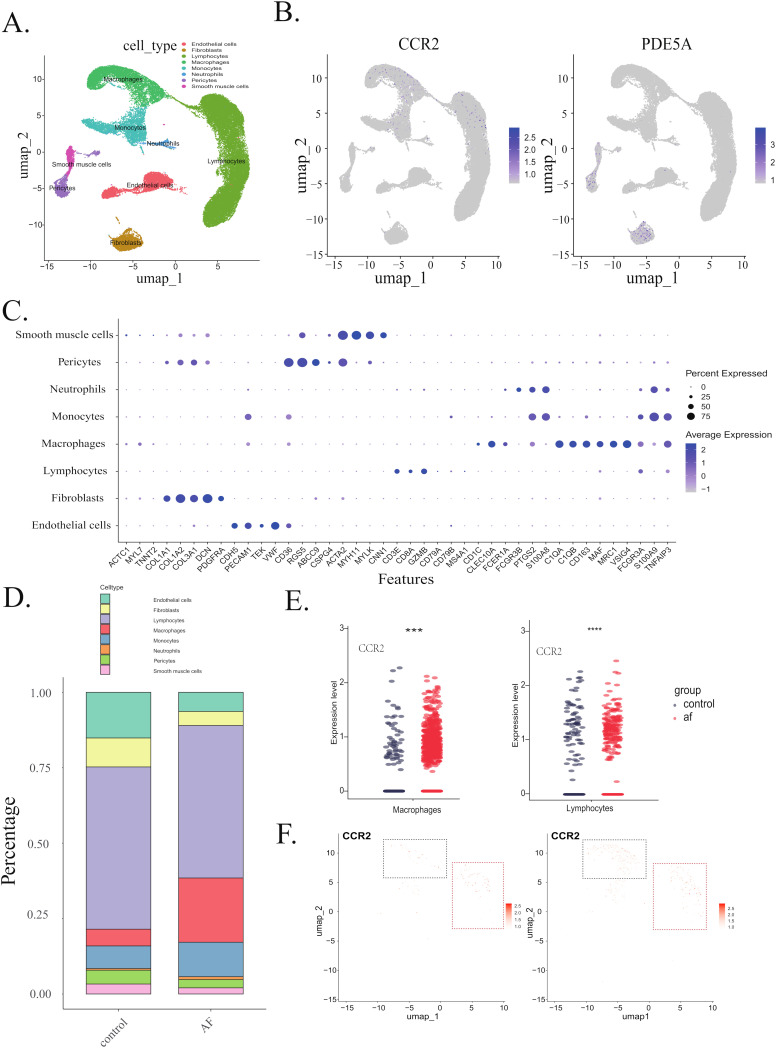
Single-cell RNA sequencing (scRNA-seq) analysis of *CCR2* and *PDE5A* expression patterns. (A) Uniform Manifold Approximation and Projection (UMAP) plot of 8 major cell types in atrial tissues. (B) UMAP plots showing the expression distribution of *CCR2* (left) and *PDE5A* (right) across all cell types. (C) Dot plot showing the expression of annotated genes across 8 cell types. (D) Bar plot showing the proportion of all cell types between atrial fibrillation (AF) and sinus rhythm (SR) groups. (E–F) *CCR2* expression in macrophages and lymphocytes between AF (right) and SR groups (left). ***P < 0.001.

Eight major cell types (endothelial cells, fibroblasts, lymphocytes, macrophages, monocytes, neutrophils, pericytes, and smooth muscle cells) were identified based on annotation for each cell subpopulation (Fig 7A). *CCR2* was found to present a higher expression level mainly in macrophages and lymphocytes, while *PDE5A* in fibroblasts (Fig 7B). To characterize the cell-type-specific expression patterns of the target genes, we performed single-cell transcriptomic profiling across 8 distinct cell populations, and visualized the results as a dot plot (Fig 7C). Differences in the proportion of all cell types were identified between the AF and SR groups, especially for an increase in macrophages (Fig 7D). Additionally, the expression of biomarkers *CCR2* and *PDE5A* in different cell types between the two groups was examined subsequently. As Fig 7E and Fig 7F display, significant differential expressions in macrophages and lymphocytes of *CCR2* can be observed. After further clustering the lymphocytes, significant differential expressions in T cells and B cells of *CCR2* can also be observed. Moreover, a significant differential expression in fibroblasts of *PDE5A* was also identified (S3 Fig).

## 4. Discussion

### 4.1. Overview of the present study

AF represents a prevalent and debilitating cardiac arrhythmia that severely compromises quality of life and elevates the risk of life-threatening complications such as stroke and heart failure [3]. For cancer patients receiving DOX-based chemotherapy, the incidence of DOX-induced AF has emerged as a critical clinical concern [31]. As for clinical practice, Toshio Kinoshita et al. [32] identified early electrocardiographic indices for predicting chronic doxorubicin-induced cardiotoxicity. However, the specific molecular mechanisms underlying DOX-induced AF remain poorly elucidated. In the present study, a multi-omics strategy integrating transcriptomics, network toxicology, single-cell analysis, and molecular docking was applied to identify key hub genes, including *CCR2* and *PDE5A*, and characterize their functions, offering new perspectives for therapeutic intervention. Molecular docking confirmed stable binding of DOX to the predicted targets *CCR2* and *PDE5A*. Finally, single-cell analysis was leveraged to resolve the cell-type-specific expression patterns of the key hub genes within the atrial tissue microenvironment. Collectively, this framework identified *CCR2* and *PDE5A* as potential therapeutic targets for DOX-induced AF and clarified their roles in specific atrial cell populations.

### 4.2. The role of *CCR2* in the DOX-induced AF

*CCR2*, a member of the C-C chemokine receptor family, serves as the main chemokine receptor of *CCL2* and plays a pivotal role in regulating immune cell recruitment and inflammation [12]. Our GSEA results revealed that *CCR2*-related genes were enriched in pathways such as antigen processing and presentation, hematopoietic cell lineage, and Th17 cell differentiation. APCs such as macrophages, may initiate and amplify an adaptive immune response. *CCR2* likely facilitates this cellular foundation by recruiting more monocytes/macrophages to the heart, thereby directly promoting a chronic inflammatory state and immune-mediated tissue remodeling. Moreover, Yang et al. demonstrated Th17/Treg ratio in serum predicts onset of postoperative AF [33].

Atrial macrophages and their monocyte-derived *CCR2* + subset expanded most among patients with AF and mitral regurgitation were also observed by Hulsmans et al. [29], which was further validated in *Ccr2* − / − mice. Corresponding with the present study, Miyosawa et al. [34] demonstrated the number of *CCR2* + monocytes/macrophages was high in left atrial appendages resected from patients with enlarged left atrial. In another study, Wu et al. [35] elucidated *CCR2* was differentially methylated and differentially transcribed in valvular atrial fibrillation (VAF) left atrial tissue.

### 4.3. The role of *PDE5A* in the DOX-induced AF

*PDE5A*, a phosphodiesterase that hydrolyzes cyclic guanosine monophosphate (cGMP), is a well-characterized regulator of cardiac contractility and vascular tone [36]. Increased expression/activity of *PDE5A* was detected in human patients with cardiac diseases [37]. Moreover, PDE5 inhibition was aproved to attenuate cardiomyocyte apoptosis and left ventricular dysfunction in a chronic model of DOX-induced cardiotoxicity [38] and diminish TGF-beta1 induced collagen synthesis of right ventricular cardiac fibroblasts in vitro induced by pulmonary artery banding [39]. *PDE5A* was found up-regulated both globally and in association with serine/threonine protein phosphatase type-1c (PP1c), which may point to a compensatory mechanism early in AF pathogenesis. A mendelian randomization study also elucidated genetically predicted PDE5 inhibition was significantly associated with a lower risk of AF [40].

In our study, the KEGG enrichment analysis showed that *PDE5A* was involved in the cGMP-PKG signaling pathway. Regarding this pathway, Huang et al. demonstrated silencing RGS7 attenuates AF progression by activating the cGMP-PKG signaling pathway [41]. In terms of GSEA analysis, leukocyte transendothelial migration was also identified as a mainly enriched in pathway in AF patients [42]. Similarly, in Zhao’s study [43], GO analysis observed the enrichment of regulating actin cytoskeleton organization in CREM-IbdeltaC-X mice with persistent atrial fibrillation. Apart from this, our study also observed that *PDE5A* is differentially expressed in fibroblasts between AF and SR patients, which was involved in atrial structural remodeling—including fibrosis and extracellular matrix (ECM) deposition [44,45]. In addition, PDE5 inhibitors have been shown to improve cardiac function and reduce arrhythmia risk in preclinical models [36], highlighting the therapeutic potential of targeting PDE5A in DOX-induced AF.

### 4.4. Cellular-level Alterations in DOX-induced AF

According to the significant results of immunoinfiltration analysis, all cell types have been elucidated associated with AF [46–48]. Single-cell analysis confirmed such change of macrophage-proportion in atria and revealed *CCR2* was differentially expression in AF patients compared to SR controls. He et al. demonstrated that the proportions of embryonic fibroblasts and actively proliferating fibroblasts were increased using the single-cell dataset (GSE197518), which comprises freshly isolated atrial cardiofibroblasts [49]. However, our study observed a decreased proportion of fibroblasts in AF patients in the Single-cell analysis. It may partly be explained that intense immune cell infiltration diluted the cellular landscape, causing fibroblasts to appear proportionally reduced, or it may reflect genuine regional differences in tissue cellularity.

### 4.5. Clinical implications and translational potential

The core therapeutic paradigm emerging from our findings involves a dual-target strategy to disrupt the pathogenic cycle of DOX-induced AF: inhibiting *CCR2* and modulating *PDE5A* activity. This approach aims to halt the progression from acute cardiotoxicity to chronic arrhythmogenic substrate by simultaneously targeting the key cellular effectors, such as macrophages and fibroblasts, within the atrial microenvironment. Translational candidates can be stratified based on their primary molecular target: (1) *CCR2/CCL2* axis inhibitors. Pharmacologic agents such as Cenicriviroc, a dual *CCR2/CCR5* antagonist, have demonstrated safety in clinical trials for inflammatory diseases [50]. Our data suggest that by blocking the *CCL2-CCR2* chemotactic axis, these compounds could specifically reduce the recruitment and pro-inflammatory activation of monocyte-derived macrophages in the atria, thereby mitigating a primary driver of atrial inflammation and subsequent structural remodeling. (2) PDE5 Inhibitors. Repurposing existing PDE5 inhibitors (e.g., Sildenafil, Tadalafil) generates a testable hypothesis that warrants evaluation in future preclinical and clinical studies, rather than representing an immediately actionable clinical strategy. Preclinical studies have shown that PDE5 inhibition attenuates DOX-induced cardiomyocyte apoptosis and left ventricular dysfunction [38]. Our findings extend this rationale to the atria, suggesting that by enhancing cGMP-PKG signaling, PDE5 inhibitors could counteract the pro-fibrotic phenotype of cardiac fibroblasts and improve endothelial function, addressing both fibrotic and vascular components of AF substrate.

Future cell-type-specific drug delivery systems to maximize on-target efficacy while minimizing systemic effects are still needed, especially crucial for immunocompromised cancer patients. Additionally, biomarker-guided therapy—using the identified hub gene panel for patient stratification could pave the way for precision medicine in the management of DOX-induced AF. Clinically, patients with elevated *CCR2* or *PDE5A* expression would be flagged as high-risk for developing AF, who might benefit most from the above findings. This would prompt clinicians to increase the frequency of ECG monitoring (e.g., weekly or bi-weekly) during DOX infusion, consider lower cumulative DOX doses or alternative anthracycline regimens, and potentially initiate targeted therapy in future clinical trials. Patients with high *CCR2* expression could be considered for use of adjunctive anti-inflammatory therapy (e.g., colchicine or *CCR2* antagonists) to reduce AF risk, while *PDE5* inhibitors (e.g., sildenafil, tadalafil) for those with high *PDE5A* expression. However, we emphasize that these interpretations are hypothesis-generating and require validation in future in vitro, in vivo, and clinical studies before any clinical application can be considered.

### 4.6. Limitations

Despite the novel findings of this study, several limitations should be acknowledged. Firstly, the transcriptomic and single-cell datasets used were obtained from public databases, which may introduce biases related to patient selection, sample processing, and clinical heterogeneity. Future studies should validate the findings in prospective cohorts of DOX-treated cancer patients to confirm the predictive value of the hub genes and the efficacy of targeted interventions. Secondly, FAERS is a passive spontaneous reporting system, which introduces inherent reporting biases, including underreporting, incomplete indication coding, and overrepresentation of certain cancer types treated with commonly reported drugs. Reports are submitted voluntarily by healthcare providers, patients, and manufacturers, and therefore the database does not capture all adverse events that occur. Moreover, this database lacks standardized and detailed clinical data, such as tumor stage, disease progression, comorbidities and detailed treatment information, which may interfere the results. Moreover, the molecular docking analysis results are needed to be verified in vitro and in vivo experiments. Thirdly, while we identified immune cell infiltration as a key feature of DOX-induced AF, the specific mechanisms by which *CCR2 +* macrophages and *PDE5A*+ fibroblasts interact to promote atrial remodeling remain unclear. Future studies should use cell co-culture models and spatial transcriptomics to dissect these cellular crosstalk pathways. Nevertheless, this study provides novel evidence and mechanistic insights into how doxorubicin (DOX) initiates and perpetuates atrial fibrillation (AF), highlighting *CCR2* and *PDE5A* as central mediators within specific cardiac cell populations, which may inform the development of precision cardioprotective strategies for cancer patients undergoing anthracycline-based therapies.

## 5. Conclusion

In conclusion, our integrated multi-omics approach identified *CCR2* and *PDE5A* as core hub genes and potential therapeutic targets for DOX-induced AF. These genes mediate atrial remodeling through cell-type-specific mechanisms involving macrophage infiltration (*CCR2*) and fibroblast-driven fibrosis (*PDE5A*), with stable binding interactions with DOX validating their direct role in DOX-induced cardiotoxicity. These findings offer a hypothesis-generating framework for understanding DOX-induced AF mechanisms and propose *CCR2* and *PDE5A* as candidate targets for further investigation. However, as this is an in silico study, all findings require validation in experimental models and clinical cohorts before any translational application.

### Use of Large Language Models, AI and Machine Learning Tools

No large language models or AI tools were used in the creation of this work.

## Supporting information

S1 Table Doxorubicin-targeted and atrial fibrillaltion-related genes. (XLSX)

S2 Table Function enrichment of doxorubicin-induced atrial fibrillation-related genes. (XLSX)

S3 Table Doxorubicin-induced atrial fibrillation-related genes in network string interactions ranked by MCC method. (XLSX)

S1 Fig FDA Adverse Event Reporting System (FAERS) analysis of adverse events related to Doxorubicin-induced atrial fibrillation (AF). (A) Flow chart showing the analysis process of the FAERS analysis. (B) Analysis of the signaling result. (C) Primary tumor type distribution based on drug indication (INDI_PT) in FAERS reports.
(DOCX)

S2 Fig Box plots comparing *KDR* and *HSP90AB1* expression levels between atrial fibrillation (AF) and sinus rhythm (SR) groups. Expression level in training (A) and validation datasets (B and C). ns = non-significant, *P < 0.05, **P < 0.01, ***P < 0.001.
(DOCX)

S3 Fig Comparison of different genes in different cell types. (A) Compare expression levels of *CCR2* in T cells and B cells between atrial fibrillation (AF) and sinus rhythm (SR) groups after further cell clustering. (B) Compare expression levels of *PDE5A* in different cell types. (C) Uniform Manifold Approximation and Projection (UMAP) plot showed *PDE5A* expression difference in fibroblasts between AF (right) and SR (left) groups. *P < 0.05, **P < 0.01, ***P < 0.001, **** P < 0.0001.
(DOCX)
