# Supplementary material for: An integrated multi-omics study of key mediators and therapeutic targets for doxorubicin-induced atrial fibrillation
Source: PLoS One. 2026 Jul 9;21(7):e0353143. doi: 10.1371/journal.pone.0353143 (PMC13349181; doi:10.1371/journal.pone.0353143)
Supplement: S1 Fig — (A) Flow chart showing the analysis process of the FAERS analysis. (B) Analysis of the signaling result. (C) Primary tumor type distribution based on drug indication (INDI_PT) in FAERS reports. (DOCX) [file pone.0353143.s004.docx]

**
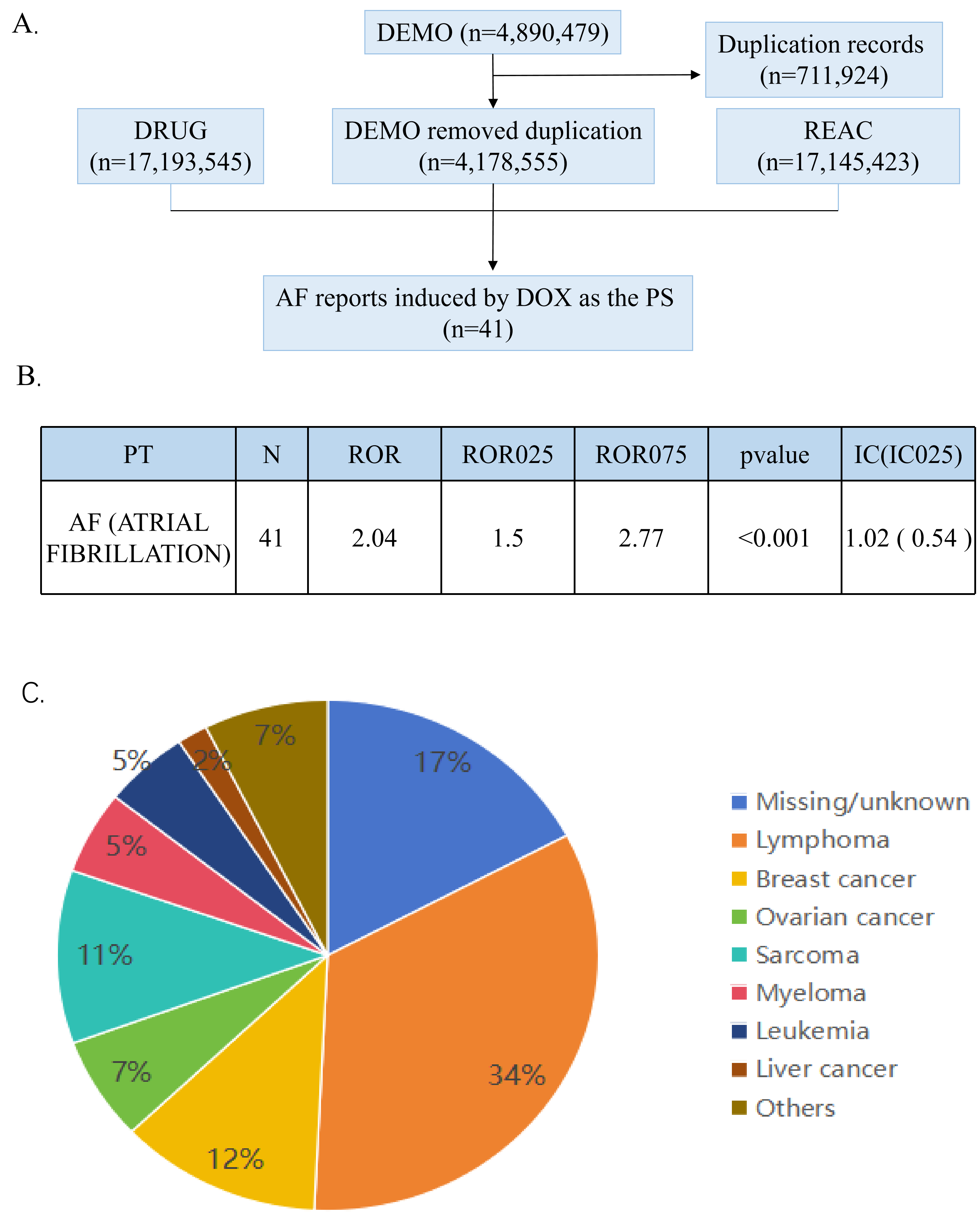
**

**S1 Fig. FDA Adverse Event Reporting System (FAERS) analysis of adverse events related to Doxorubicin-induced atrial fibrillation (AF).** (A) Flow chart showing the analysis process of the FAERS analysis. (B) Analysis of the signaling result. (C) Primary tumor type distribution based on drug indication (INDI_PT) in FAERS reports.
