# Supplementary material for: An integrated multi-omics study of key mediators and therapeutic targets for doxorubicin-induced atrial fibrillation
Source: PLoS One. 2026 Jul 9;21(7):e0353143. doi: 10.1371/journal.pone.0353143 (PMC13349181; doi:10.1371/journal.pone.0353143)
Supplement: S2 Fig — Expression level in training (A) and validation datasets (B and C). ns = non-significant, *P < 0.05, **P < 0.01, ***P < 0.001. (DOCX) [file pone.0353143.s005.docx]

**
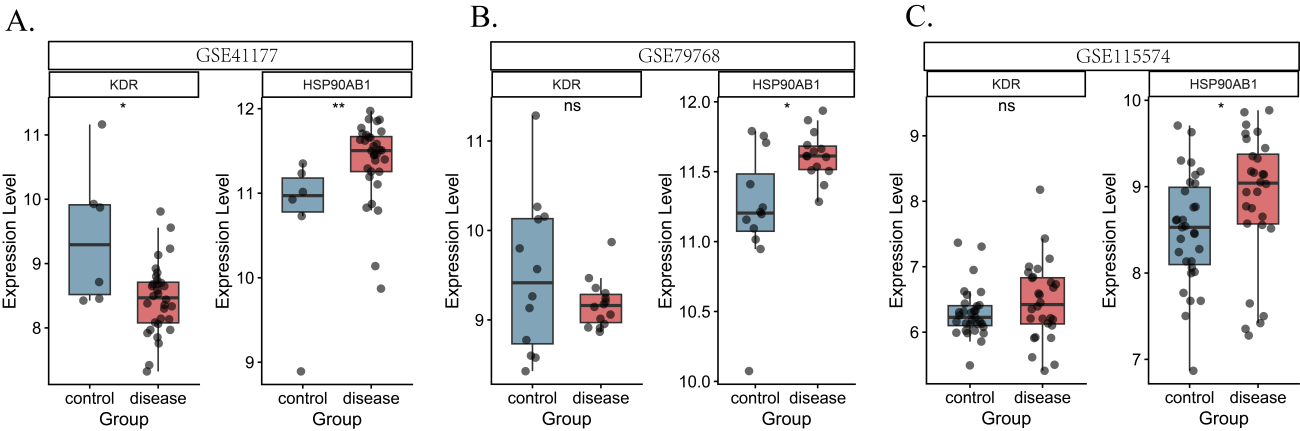
**

**S2 Fig. Box plots comparing *KDR* and *HSP90AB1* expression levels between atrial fibrillation (AF) and sinus rhythm (SR) groups.** Expression level in training (A) and validation datasets (B and C). ns = non-significant, *P < 0.05, **P < 0.01, ***P < 0.001.
