# Supplementary material for: An integrated multi-omics study of key mediators and therapeutic targets for doxorubicin-induced atrial fibrillation
Source: PLoS One. 2026 Jul 9;21(7):e0353143. doi: 10.1371/journal.pone.0353143 (PMC13349181; doi:10.1371/journal.pone.0353143)
Supplement: S3 Fig — (A) Compare expression levels of CCR2 in T cells and B cells between atrial fibrillation (AF) and sinus rhythm (SR) groups after further cell clustering. (B) Compare expression levels of PDE5A in different cell types. (C) Uniform Manifold Approximation and Projection (UMAP) plot showed PDE5A expression difference in fibroblasts between AF (right) and SR (left) groups. *P < 0.05, **P < 0.01, ***P < 0.001, **** P < 0.0001. (DOCX) [file pone.0353143.s006.docx]

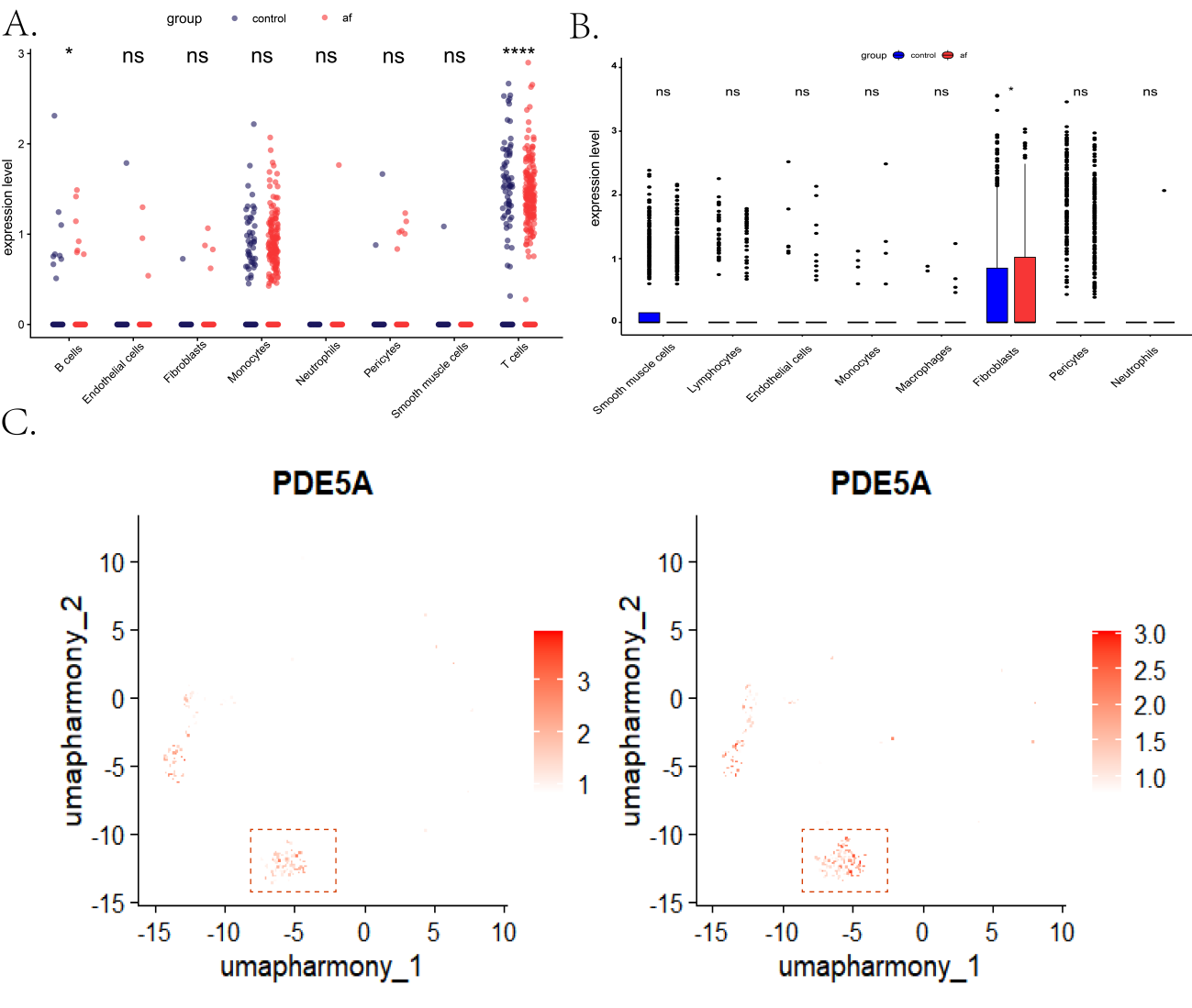


**S3 Fig. Comparison of different genes in different cell types.** (A) Compare expression levels of *CCR2* in T cells and B cells between atrial fibrillation (AF) and sinus rhythm (SR) groups after further cell clustering. (B) Compare expression levels of *PDE5A* in different cell types. (C) Uniform Manifold Approximation and Projection (UMAP) plot showed *PDE5A* expression difference in fibroblasts between AF (right) and SR (left) groups. *P < 0.05, **P < 0.01, ***P < 0.001, **** P < 0.0001.
